# Application of Modified PageRank Algorithm for Anomaly Detection in Movements of Older Adults

**DOI:** 10.1155/2019/8612021

**Published:** 2019-03-11

**Authors:** Shahram Payandeh, Eddie Chiu

**Affiliations:** Networked Robotics and Sensing Laboratory, School of Engineering Science, Simon Fraser University, Burnaby, British Columbia, Canada V5A 1S6

## Abstract

It is a well-known statistic that the percentage of our older adult population will globally surpass the other age groups. A majority of the elderly would still prefer to keep an active life style. In support of this life style, various monitoring systems are being designed and deployed to have a seamless integration with the daily living activities of the older adults while preserving various levels of their privacy. Motion tracking is one of these health monitoring systems. When properly designed, deployed, integrated, and analyzed, they can be used to assist in determining some onsets of anomalies in the health of elderly at various levels of their Movements and Activities of Daily Living (MADL). This paper explores how the framework of the PageRank algorithm can be extended for monitoring the global movement patterns of older adults at their place of residence. Through utilization of an existing dataset, the paper shows how the movement patterns between various rooms can be represented as a directed graph with weighted edges. To demonstrate how PageRank can be utilized, a base graph representing a normal pattern can be defined as what can be used for further anomaly detection (e.g., at some instances of observation the measured movement pattern deviates from what is previously defined as a normal pattern). It is shown how the PageRank algorithm can detect simulated change in the pattern of motion when compared with the base-line normal pattern. This feature can offer a practical approach for detecting anomalies in movement patterns associated with older adults in their own place of residence and in support of aging in place paradigm.

## 1. Introduction

The 2011 Census of Population counted nearly 5 million seniors aged 65 and over in Canada. Of these individuals, 92.1% lived in private households or dwellings (as part of couples or with others) while 7.9% lived in collective dwellings, such as residences for senior citizens or health care and related facilities [1]. For example, in Canada, the proportion of the elderly population increased from 8% to 14% from 1971 to 2010 and is projected to represent 23-25% of the total population by 2036. The percentage of elderly living in special care facilities reaches 30% by the age of 85. British Columbia's share of seniors 65 and older was 15.7 per cent, above the Canadian national average of 14.8% in 2011 [2]. These trends are also shared with similar statistics across the globe. A report from the UN indicates that “between 2015 and 2030, the number of people aged 60 years or over is projected to grow by 56% more than double its size in 2015” [3].

Being able to monitor movements and activities of older adults through various available sensing modalities and more importantly being able to effectively analyze and display the sensed information can benefit both the care-giving personnel and family members [4]. Such sensor deployment in the smart living environment can also allow development of various movements and activity monitoring systems. Sensor deployment can also create a unique opportunity in finding various frameworks for detecting anomalous movement patterns and behaviors which deviate from normal patterns of movements and activities. Advances in sensors, smart homes, and elderly robotics have allowed a low-cost and consistent stream of sensor data of daily life environments which could be used to track movements and detect anomalies.

Being able to detect any changes in long-term monitoring of movements and activities patterns can assist in identifying onset of anomalies, which can potentially provide valuable insights for the physicians about the risk of frailty [5]. In general, it is a complex task to determine the degree of frailty associated with the movements and activities of older adults. This would, for example, depend on the amount of actual information that can be collected from the deployed sensors in the living environment of the elderly. In addition, without assistance of physicians or care-giving personnel, it is also challenging to determine normal patterns of movements of older adults.

For the aging population, many would prefer to spend a large proportion of their time at their private home for as long as they can. In support of this living arrangement, various notions of smart living environment for the elderly have been proposed. For example, [6] proposed an easy to install smart home system. Smart home kits are capable of activity recognition with 84% accuracy. The smart homes were rated on how easily they can be deployed by several participants with varying technological backgrounds. The core component of such a living environment is the availability of various motion and activity sensors to be deployed at various locations of the living environment. A hidden Markov model (HMM) to analyze and model daily activities using data collected over a period of 8 days was proposed in [7]. Motion data is converted into the state vector and used in a HMM to reconstruct an activity model. An approach for outlier detection in activity record was proposed in [8] where outliers were defined to be activity errors. Using machine learning models trained with activity data with no errors (i.e., normal patterns of activities), they were able to detect and classify activities with errors. Sensor data from 580 participants from two smart homes were used and labeled externally with activities and errors. These activities were evaluated through a base-line algorithm created by only sensor counts for later comparison. The activity data was trained with a one class support vector machine. A set of features which can be used for a screening system to help diagnose dementia was proposed in [9]. Movement data was utilized to train a Bayesian network machine learning model to classify patients. Publicly available data was used with activity annotations and scores. K-repeating substrings were used to extract features from movement trajectories, which are used as an input into the Bayesian network. Sensor data from smart homes combined with machine learning are utilized to classify people with Parkinson disease and mild cognitive impairment based on behavioral patterns doing a set of daily life activities [10]. 260 participants were screened for neurological disorders and other medical anomalies. 84 of the 260 participants were chosen to perform activities in a smart home with two wearable sensors, one on the dominant arm and one on the dominant side ankle. Sensor events were collected from the smart home and annotations were added automatically with their annotation system. Features were extracted from the data, and machine learning algorithms decision trees, Naïve Bayes, random forest, support vector machines, and adaptive boosting were used with the extracted features. It was found that all machine learning algorithms did better than random classification in distinguishing healthy patients from Parkinson disease patients.

PageRank is an algorithm used by Google Search to rank web pages in their search engine results. PageRank works by counting the number and quality of links to a page to determine a rough estimate of how important the website is. The underlaying assumption is that more important websites are likely to receive more links from other websites [12]. Given a typical living environment of an older adult consisting of rooms and connected hallways, this paper presents a modified PageRank algorithm that ranks rooms/hallways which are visited the most by older adults for a period of observation. This paper further evaluates to see whether deviations in the recorder data could cause a change in the ranking of the rooms defined by the modified PageRank algorithm. This approach is an alternative algorithm for detecting anomalies in movements and activities of older adults in comparison with the reviewed literature which are mostly based on HMM, Bayesian framework, or machine learning algorithms. In our study, sensor data was obtained from publicly available datasets [6] and preprocessed and clustered into designated rooms.

The paper is organized as follows: Section 2 presents some background motivation; Section 3 presents data processing and construction of directed graph using the supplied dataset; Section 4 presents details of the modified PageRank algorithm and its application to the directed graph representation of movements patterns; Section 5 presents some experimental analysis on how the PageRank can be used for anomaly detection; and Section 6 presents discussions and concluding remarks.

## 2. Background and Motivation

**Figure 1:**
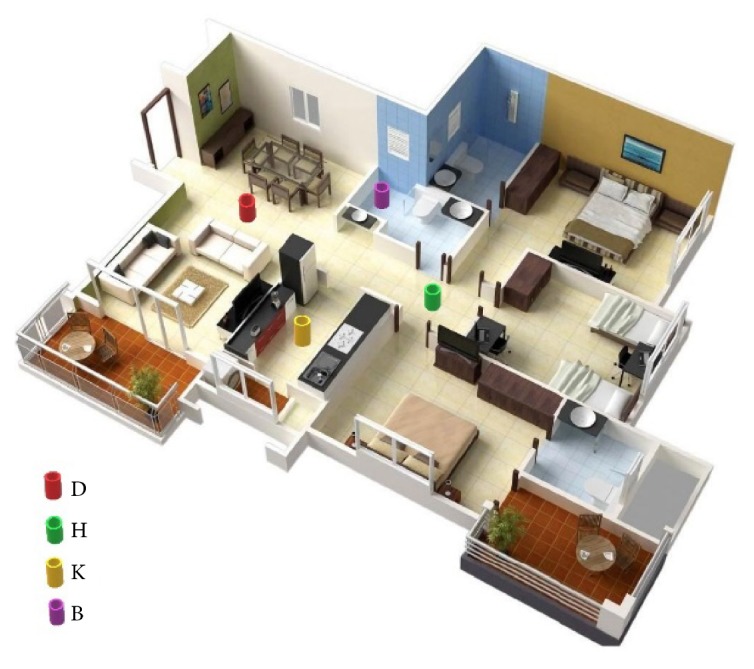
A representative example of living environment of an older adult with definition of various key markers. The key nodal sensing points are dining room (D); hallway (H); kitchen (K), and bathroom (B). Figure 1 is reproduced from Payandeh, 2018 [11] (under the Creative Commons Attribution License/public domain).

Figure 1 shows a representative example of the living environment of an older adult. This Figure also shows an example of representative key movement sensing points depicted as cylindrical objects (i.e., a representative of the locations of motion sensors associated with a room). For example, the red, green, yellow, and purple indicators correspond to dining room (D), hallway (H), kitchen (K), and bathroom (B). Following the general description of motion of older adults [11] and referring to Figure 1, we also define the first level of detail in monitoring of movements on how often the key sensing points are visited by an older adult given a time window of observation. Level two would be details representing the motion trajectory of the older adult between each of the key sensing nodes, i.e., say between nodes H and D. The third level would be the gait patterns of elderly as she/he moves between any of these key nodal points (for example from key nodal station H (the green cylinder) to K (the yellow cylinder)). Similarly, additional key location indicators can be introduced in each of the rooms which can be used to further characterize the movements and activities in each of the rooms.

**Figure 2:**
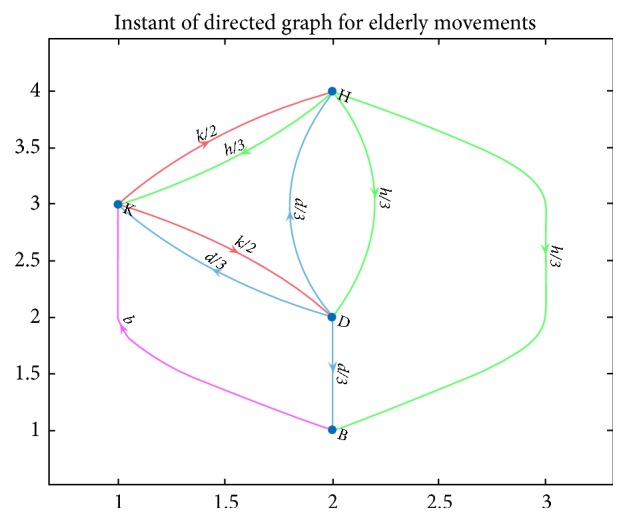
An example of weighted directed graph representation for the reference key nodal sensing points shown in Figure 1.  Figure 2 is reproduced from Payandeh, 2018 [11] (under the Creative Commons Attribution License/public domain).

In general, and referring to Figure 1, reference key nodal sensing points in an older adult's living environment can also be represented as nodes in a directed movement graph. The edges of this graph represent the directions of motion between each of the nodes. For a given time window of observation, one can also collect and assign a probability distribution that the elderly can visit a selected node of the graph from any other node. Such a probability distribution can be described as a weight vector for each of the out-going edges of the directed graph.  Figure 2 shows an example of such graph structure for the reference nodal description of Figure 1. For example, considering the hallway node H where the elderly is detected, there is an equal probability (say (h/3)) that the elderly can go to the kitchen, dining room, or bedroom.

The directed graph of Figure 2 can represent a sampled event of the probability distribution of movements given a period of observation. For example, when an elderly is detected at an instant of observation (e.g., in the evening) at a key sensing node associated with the hallway (H), an equal initial probability can be assigned to the elderly for either going to the bathroom (B), the kitchen (K) or the dining room (D). Similarly, as observed from the graph of Figure 2, there is also an equal probability for the elderly to go from kitchen (K) to the dining room (D) or to the hallway (H).

The weighted directed graph of Figure 2 can also be casted into a Markov transition matrix (stochastic matrix) like the following representation:

(1) $$\begin{array}{c} A=\left[\begin{array}{cccccc} H & K & D & B & \;|\; & \\ - & - & - & - & \;|\; & -\\ 0 & \frac{1}{2} & \frac{1}{3} & 0 & \;|\; & H\\ \frac{1}{3} & 0 & \frac{1}{3} & 1 & \;|\; & K\\ \frac{1}{3} & \frac{1}{2} & 0 & 0 & \;|\; & D\\ \frac{1}{3} & 0 & \frac{1}{3} & 0 & \;|\; & B \end{array}\right]. \end{array}$$

Columns of the matrix represent the probability distribution of older adult visiting the other rooms through row association. For example, when the older adult is at B, there is a 100% chance that K will be visited. Given such transition matrix* A*, it can further be utilized to predict a long range behavior of an older adult similar to method used in [13]. For example, let us assume that initially the elderly has an equal probability distribution *x*_0_ to be at either of the key sensing points and equal probability to visit the corresponding connected linked rooms, i.e., *x*_0_ = 1/*n*; *n* = 4; *x*_0_ = 1/4 where* n* is the number key nodal sensing points. Using the notion of Markov chain and the definition of the transition matrix A, the chained probability distribution of the elderly being in various rooms over instances of *n* observation can be estimated as

(2) $$\begin{array}{c} x_{1}=Ax_{0}\\ x_{2}=Ax_{1}\\ \vdots \\ x_{n+1}=Ax_{n}\\ ↓\\ x_{n}=A^{n}x_{0}. \end{array}$$

Such long-term distribution of behavior can also be determined through eigenvalues and eigenvectors computation of the transition matrix A.

Another utilization of the Markov transition matrix A for predicting the long-term behavior of the elderly is through an application of the basic PageRank algorithm [14]. PageRank is an algorithm used by Google Search to rank web pages in their search engine results. PageRank works by counting the number and quality of links to a page to determine a rough estimate of how important the website is. The underlaying assumption is that more important websites are likely to receive more links from other websites. For example, for an instant of observation, say (*n* + 1), we are going to define the probability an older adult being at a given room, say *R*_*i*_, defined as *P*_*n*+1_(*R*_*i*_) to be equal to sum of the probabilities of the older adult being in the rooms pointed to the room *R*_*i*_ at the previous instant, namely, *P*_*n*_(*R*_*j*_), divided by the number of edges pointing out from room *R*_*j*_, namely, *C*(*R*_*j*_), or

(3) $$\begin{array}{c} P_{n+1}\left(\;R_{i}\;\right)=\underset{R_{j}}{\sum }\frac{P_{n}\left(R_{j}\right)}{C\left(R_{j}\right)}. \end{array}$$

Given the previously defined initial probability distribution (i.e., equal probability that the older adult can be detected in any of the key observation points), iteratively we can compute the probability distribution at the next cycle where the older adult can be, for example, the hallway (H) as *P*_1_(*R*_*H*_) = (1/4)/(2)+(1/4)/(3) = (2.5)/(12) and the kitchen (K) as *P*_1_(*R*_*K*_) = (1/4)/(3)+(1/4)/(3)+(1/4)/(1) = (5)/(12).

Table 1 summarizes the results of these transitions for the two cycles of prediction.

As it can be seen from Table 1, the dining room (D) is ranked higher than other rooms. This also implies that, for a given Markov transition matrix, the steady-state probability distribution solution for all key nodal sensing points in the monitoring area can be determined. This indicator can be used to rank the statistical preference of the older adult during an observation period (a nominal period which the future distribution patterns can be compared with). Another consequence of the Markov matrix is that one of its eigenvalues is always equal to one and all the other eigenvalues will have absolute values less than one. One interpretation of the eigenvector of the Markov matrix associated with eigenvalue of one is based in Perron-Frobenius contribution which states that such eigenvector corresponds to the equilibrium distribution of the matrix over its states as the number of sequence of observation becomes large.

## 3. Application of a Modified PageRank Algorithm

PageRank is a method for rating web pages objectively and mechanically, effectively measuring the human interest and attention devoted to them [15]. In order to measure the relative importance of web pages, PageRank proposed a method for computing a ranking for every web page based on the graph of the web.

At each iteration of PageRank, nodes give their current PageRanking across their links and gain PageRanking from other nodes that link into the nodes. All the nodes' outbounding and inbounding links can be shown in a matrix representation, a transition matrix. A version of the PageRank algorithm can be written as [14]

(4) $$\begin{array}{c} R\left(\;u\;\right)=\underset{v\in B_{u}}{\sum }\frac{R\left(u\right)}{N_{v}}, \end{array}$$

where *R*(*u*) is the rank of node* u*, *N*_*v*_ is the total number of links out of* u, *and *B*_*u*_ is the set of all nodes that link to* u*.

In graph representations, there exist sink nodes which only have inbound links, i.e., dead-end nodes, or disconnected components. A form of the general PageRank algorithm can be modified to account for such conditions, i.e.,

(5) $$\begin{array}{c} R\left(\;u\;\right)=\frac{1-d}{N}+d\underset{v\in B_{u}}{\sum }\frac{R\left(u\right)}{N_{v}}, \end{array}$$

where (1 − *d*)/*N* is the condition factor such that an older adult will ignore the transition probabilities of the current state defined by the Markov matrix and instead randomly jump to another node.

As it was stated, the underlying transition matrix is the core of the PageRank algorithm. Thus, PageRank is akin to a Markov chain. A Markov chain determines a sequence of states through transition probabilities between one state to another, and the probability of the next state depends only on the previous state. The states can be hidden and instead have emission probabilities to observable data, which leads to the hidden Markov model. In PageRank's case, the states are the pages, where a given state is true when that page is visited, and the transition probabilities for a given state to other states are equally split between the outlinks for that given state. In the case study of this paper, we have utilized a modified version of the PageRank algorithm. Here, instead of splitting the PageRank of nodes equally among its out links, a transition weight for each nodes' outlinks is statistically determined from the dataset. The number of events from one room to another was counted then normalized for each room to represent the probability of going from one room to another. Then, PageRank is run with the new weights. A version of the PageRank algorithm which was utilized in this paper is defined as

(6) $$\begin{array}{c} P\left(\;A\;\right)=\frac{1-d}{N}+d\underset{v\in B_{u}}{\sum }P\left(\;u\;\right)*M_{u}^{T}, \end{array}$$

where P(·) is the PageRank of (·), where (·) is a node in the graph, d is the damping factor, N is the number of nodes, M is the right stochastic matrix for the rooms, and *M*_*u*_^*T*^ is the column of M where u indexes. We have used a damping factor of 0.99 throughout our studies.

### 3.1. Data Collection and Preprocessing

Datasets from the Center for Advanced Studies in Adaptive System (CASAS) were examined [6]. The CASAS project uses smart home environments for research and data collection. They have made publicly available a number of datasets collected with their smart home environments. The datasets contained sensor event data collected from smart homes with at least one resident across a period of a few days to months.

**Figure 3:**
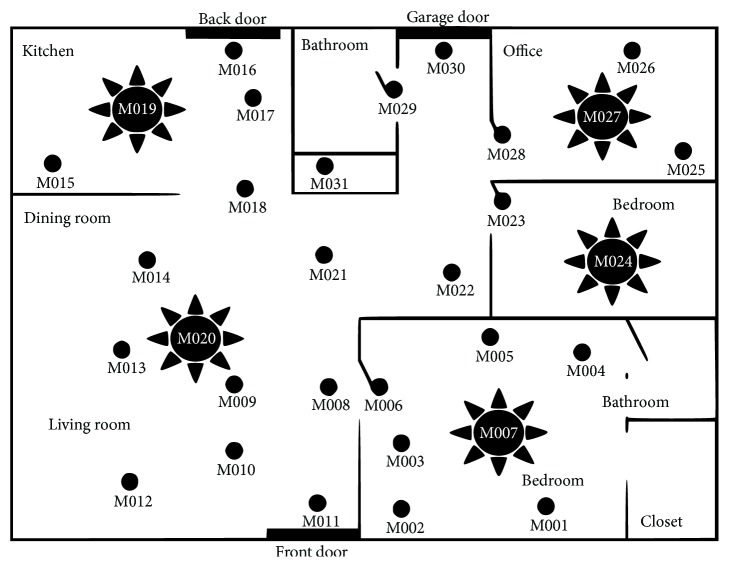
An example of floor plan with sensor layout used in CASAS. Each MXXX ID is a motion sensor [6].

**Figure 4:**
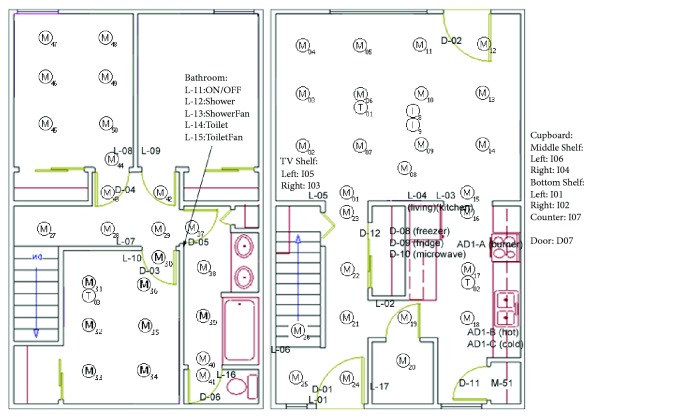
Floor plan with sensor locations used in this study. M indicates motion sensors and T indicates temperature sensors [6].

The dataset we have chosen had sensor data collected across three days for two residents, with some extended over to the following day. The first day (2008-06-24-25) went from 7:49:21 PM to 2:41:42 AM on the 25th, the second day (2008-06-29) went from 6:13 AM to 1:56 AM on the 30th, and the third day went from 6:04 AM to 1:48 AM on July 1^st^. The sensors types include motion, temperature, and water sensors and their locations were shown on a floor plan image as seen in Figures 3 and 4.

For this study, only motion sensor events were utilized. The recorded sensor event format was structured as “date time SensorID-residentID”. The two resident's sensor activation can be further filtered to extract data associated with a single resident. We have only used data associated with a single resident. A sample of the data is shown in Table 2.

**Figure 5:**
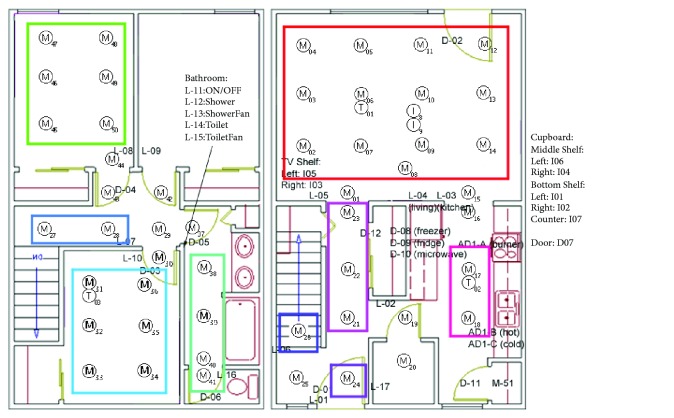
An example of sensor clusters where different color indicates different regions or rooms in the monitoring area.

To obtain room transitions, sensor data from multiple sensors in the same room were clustered, forming a single cluster representation of each room as shown in Figure 5. Sensors that were on the border between multiple rooms were removed to improve accuracy in recording the transitions between each room. With these room events, a room-to-room edge list is created, with an interval that indicates how long the subject stayed in the starting room. A small interval indicated rapid room changes. Intervals that lasted two seconds or less were filtered to reduce false positives from sensitive sensors. Additionally, impossible room edges such as edges going from the 1^st^ floor to the 2^nd^ floor without passing through the stairs were also filtered.

## 4. Activity Graph

**Figure 6:**
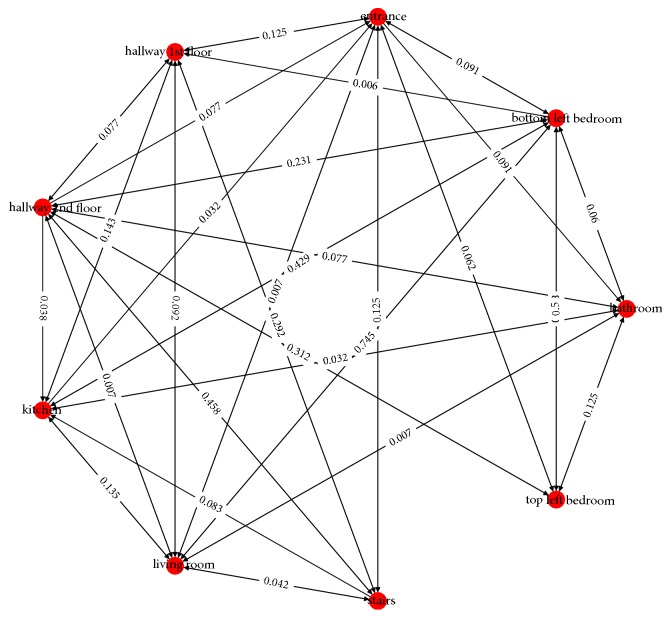
The directed graph of the unfiltered data.

Given the room-to-room edge list, and a room vertex list, a directed graph can be produced from the data, seen in Figure 6.

**Figure 7:**
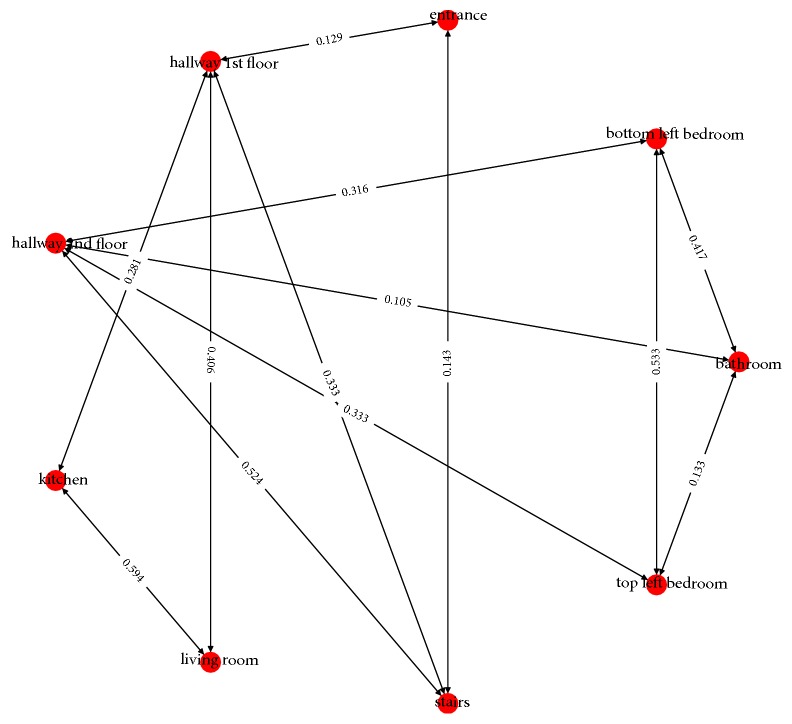
A simplified directed graph after applying the proposed filtering of data.

The graph contains a number of impossible edges with a low transition probability, indicating sensor error, thus creating more edges than should be possible. To simplify the graph and correct for possible sensor errors, a number of ad hoc filters were further utilized. First, sensors which are located at the connecting boundaries between the rooms were removed and only considering sensors which are within the center area of the room cluster. Next, if the interval between the current event and the next event is small, then those events were removed (Figure 7). For example, in this study, all events that lasted less than 3 seconds were removed.

## 5. A Study for Anomaly Detection

Our hypothesis is that anomalous activity in movement pattern could be detected by observing changes from the nominal pattern defined by in the nominal PageRank. The nominal PageRank is obtained from data which can be considered and used as a basis for the comparison. For example, Table 3 shows the ranking of the rooms determined by PageRank for 2 days observation window.

To evaluate this hypothesis, a series of simulated movements and activities are introduced in the nominal data. To accomplish this, a small block of the transition matrix was modified, indicating anomalous activity, and then the PageRank algorithm was implemented with the modified transition matrix. In the first experiment, the modified transition matrix was to emulate the increased probability that the subject used the bathroom. As such, the objective is to observe if the PageRank algorithm can identify such simulated anomalies in the movement patterns. This could represent a person that suddenly has some sort of need to more frequently go to the bathroom.

**Experiment 1 .**

In bottom left bedroom's transitions, split 0.1 (out of 0.25) from hallway 2^nd^ floor and 0.1 (out of 0.333) from top left bedroom, and add it to bathroom (0.417 to 0.617).

*Result. *Bathroom ranked jumped to 3^rd^ from 8^th^ (Table 4).

**Experiment 2 .**

In top left bedroom's transitions, split 0.1 (out of 0.333) from hallway 2^nd^ floor and 0.1 (out of 0.533) from bottom left bedroom, and add to bathroom (0.133 to 0.333).

*Result. *Bathroom went from 8^th^ to 6^th^. (Table 5)

**Experiment 3 .**

In 2^nd^ floor hallway's transition, split 0.07 from bottom left bedroom (out of 0.316), 0.07 from stairs (out of 0.368), 0.07 from top left bedroom (out of 0.211), and add to bathroom (0.105 to 0.315).

*Results. *Bathroom jumped to 3^rd^ from 8^th^. (Table 6)

In all experiments, increasing the number of bathroom visits by increasing the weight of bathroom edges made the bathroom rise in PageRank.

## 6. Discussion and Conclusions

The study of this paper demonstrates that various forms of the PageRank algorithm can be explored as a tool in order to identify and detect anomalous movement patterns of older adults in a home environment. Such detection can further assist in detecting physical problems like incontinence, or mental problems, like Alzheimer's disease. The method of this paper offers an alternative approach in comparison with the literature for detecting anomalies in movement patterns of older adults which is based on the proven PageRank methodology. One of the challenges of the proposed framework is the identification of what can be considered as normal patterns of movements or activities. For example, for the first case study, it can be observed that the algorithm was able to detect that the visits to the bathroom have been increased in comparison to what was defined previously as a normal ranked pattern (Table 5). Another challenge is the definition of a feasible window of observation corresponding to the start and the duration of the data collection for creation of a basis for future comparison. These base-line data are person specific which can be collected with the help of care-givers or family physicians. The proposed clustering of the sensors which uses their location identifier associated with a particular room can be applied to rank activities of an older adult within a specific room. In this case, sensors associated with various locations within a room can be used to further rank the activities within a room. The method of this paper is concerned only with the detection of movements between various key nodal sensing points. For cases where the study of trajectory of the movements is of importance, other types of sensors can be utilized for determining the tortuosity of the path [16]. In addition, for the cases where the configuration of gait and its pattern are of importance, other ambient sensing modalities such as depth sensors can be utilized [17].

## Figures and Tables

**Table 1:** An example of two-cycle transition of Markov matrix using the basic PageRank algorithm associated with Figure 2. Table 1 is reproduced from Payandeh, 2018 [11] (under the Creative Commons Attribution License/public domain).

	n=0	n=1	n=2
H	1/4	2.5/12	3.33/12

K	1/4	5/12	3.66/12

D	1/4	2.5/12	5.33/12

B	1/4	2/12	1.66/12

**Table 2:** Sample of sensor data log file.

*Date*	*Time*	*SensorID-residentID*
6/29/2008	06:12:58	M47-1

6/29/2008	06:13:01	M48-1

6/29/2008	06:13:04	M48-0

**Table 3:** Ranking of the connected rooms associated with an example of normal movements and activities.

*Ranking*	*PageRank 2008-06-29*
1	bottom left bedroom

2	hallway 1st floor

3	kitchen

4	living room

5	hallway 2nd floor

6	top left bedroom

7	stairs

8	bathroom

9	entrance

**Table 4:** Results of the first experiment.

*Ranking*	*PageRank 2008-06-29*	*Modified PageRank 2008-06-29*
1	hallway 1st floor	bottom left bedroom

2	bottom left bedroom	hallway 1st floor

3	kitchen	bathroom

4	living room	kitchen

5	hallway 2nd floor	living room

6	top left bedroom	hallway 2nd floor

7	stairs	top left bedroom

8	bathroom	stairs

9	entrance	entrance

**Table 5:** Results of the second experiment.

*Ranking*	*PageRank 2008-06-29*	*Modified PageRank 2008-06-29*
1	hallway 1st floor	bottom left bedroom

2	bottom left bedroom	hallway 1st floor

3	kitchen	kitchen

4	living room	living room

5	hallway 2nd floor	hallway 2nd floor

6	top left bedroom	bathroom

7	stairs	top left bedroom

8	bathroom	stairs

9	entrance	entrance

**Table 6:** Results of the third experiment.

*Ranking*	*PageRank 2008-06-29*	*Modified PageRank 2008-06-29*
1	hallway 1st floor	bottom left bedroom

2	bottom left bedroom	hallway 2nd floor

3	kitchen	bathroom

4	living room	hallway 1st floor

5	hallway 2nd floor	kitchen

6	top left bedroom	living room

7	stairs	top left bedroom

8	bathroom	stairs

9	entrance	entrance

## Data Availability

Previously reported sensor data were used to support this study and are available at http://casas.wsu.edu/datasets/. These prior studies (and datasets) are cited at relevant places within the text as reference [6].
